# Elevated Cardiovascular Risks among Postmenopausal Women: A Community Based Case Control Study from Nepal

**DOI:** 10.1155/2017/3824903

**Published:** 2017-05-02

**Authors:** Bashu Dev Pardhe, Sumitra Ghimire, Jyotsna Shakya, Sabala Pathak, Shreena Shakya, Anjeela Bhetwal, Puspa Raj Khanal, Narayan Prasad Parajuli

**Affiliations:** Department of Laboratory Medicine, Manmohan Memorial Institute of Health Sciences, Kathmandu, Nepal

## Abstract

Cardiovascular disease (CVD) is one of the leading causes of death worldwide which is more prevalent in women after menopause. Hormonal changes associated with menopause are accountable for dyslipidemic pattern that causes CVD and associated complications. Therefore, the present study was commenced to compare the lipid profile in pre- and postmenopausal women. A descriptive cross-sectional study was conducted at Manmohan Memorial Institute of Health Sciences (MMIHS) from February 2016 to July 2016. A total of 260 fasting samples were collected from healthy women, 130 from premenopausal and 130 from postmenopausal women, and analyzed for Total Cholesterol (TC), Triacylglycerol (TAG), High Density Lipoprotein Cholesterol (HDL-C), and Low Density Lipoprotein Cholesterol (LDL-C) as per the guideline provided by the reagent manufacturer (Human, Germany). All the parameters were analyzed by Stat Fax 3300 semi auto analyzer. TC, TAG, HDL-C, and LDL-C were highly significantly increased in postmenopausal women when compared to premenopausal women. LDL/HDL ratio was significantly elevated in postmenopausal women than in premenopausal women. BMI was significantly positively correlated with TC and TAG in both pre- and postmenopausal population and it was positively correlated with HDL-C in premenopausal population while negatively correlated in postmenopausal population. Since more of the atherogenic lipid parameters are increased in postmenopausal women, they appear to be more prone to have CVD and associated complications in near future. Hence, it is mandatory to monitor and manage dyslipidemic pattern in every woman experiencing menopause.

## 1. Background

Cardioprotective effect on premenopausal women is believed to be imposed by adequacy of endogenous estrogen level produced during menstrual cycle. This could be the possible reason for declined rate of coronary heart disease in fertile women than men [1]. However, by the end of reproductive life ovaries fail to produce significant amount of estrogen instigating postmenopausal women more prone to disease associated with estrogen deficiency like heart diseases, osteoporosis, and dyslipidemia [2, 3]. Hormonal changes after menopause such as low plasma estrogen level and elevated Luteinizing Hormone (LH) and Follicle Stimulating Hormone (FSH) level have significant effect on plasma lipid and lipoprotein metabolism resulting in ultimate cardiac related disorders [4, 5].

Estrogen shows cardioprotective effect by maintaining high level of HDL-C and low level of LDL-C and TAG. Mass clearance of LDL-C from the plasma results probably from accelerated conversion of hepatic cholesterol to bile acids and increased expression of LDL receptors on cell surfaces. Increase in production of apolipoprotein A-I and decrease in hepatic lipase activity facilitate increase of HDL-C [6]. Estrogen causes hepatic expression of apoprotein gene; consequently it affects lipid and lipoprotein metabolism [1].

Such cardio defensive effect is lost after menopause driving postmenopausal women towards high risk of debilitating and often fatal complications of cardiovascular disease (CVD) [7]. Estrogen hormone regulates cellular level directly by mRNA production for specific protein including proteins for lipid metabolism such as lipoprotein lipase (LPL) and hormone sensitive lipase (HSL) in adipose tissue. Estrogen also has indirect action on adipose tissue by stimulating release of other hormones such as growth hormone (GH), catecholamine, and glucagon which increase activity of HSL. 17-beta-estradiol (a major circulating form of estrogen) regulates the rate of synthesis of structural apolipoproteins for VLDL and HDL in liver. It reduces the rate of apoB-100 synthesis, thereby reducing the VLDL concentration which is risk factor for atherosclerosis. Conversely, it enhances the rate of apoA-I and apoA-II synthesis, thereby increasing HDL concentration which is atheroprotective. HDL containing apoA-I and apoA-II helps to degrade cholesterol from VLDL and chylomicron by reverse cholesterol transport from peripheral tissue to liver [8].

TC and TAG contribute major circulating lipid in our body, while chylomicron, VLDL, IDL, LDL, and HDL are the lipoproteins functioning as vehicle for cholesterol transport. Cholesterol content in VLDL, IDL, LDL, and HDL determines the total plasma cholesterol. LDL conveys endogenous cholesterol from the liver to the peripheral tissues. Under certain circumstances, LDL deposit cholesterol in the intimal layer of the arteries, thereby initiating the atherosclerotic process. HDL functions as atheroprotective since it has reverse cholesterol transport activity, thereby removing excess cholesterol from cells, including cholesterol engulfed macrophages in atherosclerotic lesions, and transporting it into the liver for hepatic excretion through bile acid. Onset, progression, and complications of atherosclerotic plaque are determined by the balance between these two lipoproteins [9].

A fatty streak lesion is produced with an accumulation of cholesterol esters in the intimal layer of the arteries, indicating the initiation of atherosclerosis. The fatty streak may progress to atherosclerotic plaque if the process continues over time. If the plaque continues to grow it may block the vessel or form thrombosis and lead to further complication of ischemic disease like ischemic stroke, Coronary Heart Disease, or Peripheral Artery Disease [9].

An estimated 17.5 million people died from CVD in 2012, representing 31% of all global deaths. Over three quarters of CVD death occur in low and middle income countries [10]. According to American Heart Association report (2002), after menopause 70% of women develop cardiovascular disease and 30% develop osteoporosis in USA [3].

Dyslipidemia, especially hypercholesterolemia, is the major risk factor of CVD. Cardiovascular disease is the most vulnerable health problem prevailing in every corner of world including Nepal. In Nepal, women cover beyond 50% of population among which more of the population are over the age of 50 years. Specially, this group of population lacks physical activities due to their retired life; in addition, consumption of unhealthy food makes them more prone to suffer from atherogenic effects. This is the mean age for menopause suggesting that more of the Nepalese women are probably at risk of developing complications associated with cardiovascular disease which is the matter of concern [11].

Since estrogen plays decisive role in lipid and lipoprotein metabolism, it is indispensable to monitor lipid profile in postmenopausal women who tend to have diminished estrogen level. Hence, the present study was undertaken to compare serum level of TC, TAG, HDL-C, and LDL-C in pre- and postmenopausal women

## 2. Methods

A descriptive cross-sectional study was conducted during the period of six months among the female population of Dholahity community, Lalitpur, Nepal. A total of 260 overnight fasting blood specimens (5 ml) were collected from healthy women, 130 from pre- and 130 from postmenopausal women. Serum samples were separated for the analysis of Total Cholesterol (TC), Triacylglycerol (TAG), High Density Lipoprotein Cholesterol (HDL-C), and Low Density Lipoprotein Cholesterol (LDL-C) as per the guideline provided by the reagent manufacturer (Human GmBh, Germany). All the parameters were analyzed by Stat Fax 3300 semi auto analyzer in the Department of Laboratory Medicine, Manmohan Memorial Institute of Health Sciences.

### 2.1. Inclusion and Exclusion Criteria

Apparently healthy women aged between 22 and 85 years were included in the study. Women who undergone menopause due to hysterectomy or cessation of periods other than by a natural cause, women on hormone replacement therapy (HRT), women having irregular menses, thyroid patients, pregnant and lactating women, diabetic women, heavy smokers, and alcoholic women were excluded.

### 2.2. Ethical Approval

Written permission was taken from Institutional Review Committee (IRC) of Manmohan Memorial Institute of Health Sciences (MMIHS) and informed consent was taken from each individual.

### 2.3. Data Analysis

Data were analyzed using SPSS version 20.0 and Microsoft Excel 2013. Student's *t*-test was used to analyze differences in lipid profile between pre- and postmenopausal groups. Pearson correlation coefficient was used to evaluate the association of BMI with lipid parameters.

## 3. Results

The study was carried out in 260 healthy women; among them 130 were premenopausal and 130 were postmenopausal. The average age of premenopausal group was 35.96 ± 8.6 and that of postmenopausal group was 64.5 ± 11.1. Serum TC, TAG, HDL-C, and LDL-C levels were found to be statistically significantly increased in postmenopausal women compared to premenopausal women (*P* value < 0.05) (Table 1).

The atherogenic indices TC/HDL and TAG/HDL were found to be increased but not significantly in postmenopausal women compared to premenopausal women. However, LDL/HDL ratio was found to be statistically significantly increased in postmenopausal women than in premenopausal women. We found that 18.4% of postmenopausal women were under cardiovascular risk as defined by TC/HDL indices greater than 5 (Table 2).

BMI was significantly positively correlated with TC and TAG in both pre- and postmenopausal women. Besides, BMI was found to be negatively correlated with HDL-C in postmenopausal women while positively correlated in premenopausal women (Table 3).

## 4. Discussion

Low and middle income countries are in the peril of noncommunicable disease which is almost half of the total disease burden. Cardiovascular disease solely attributed for 30% of death worldwide in 2005 only [12]. Coronary Artery Disease (CAD) among men is more prevalent than women up to the age of 50 years, but then the incidence is overturned afterwards causing increased prevalence of CAD in women [13]. Most probable reason behind this could be the loss of cardioprotective effect of estrogen which is slowly downturned in women after menopause. Therefore, it necessitates screening of every women undergoing menopause for early diagnosis and prevention of CVD especially in the economically poor countries like Nepal where more than half of the population are women.

This time framing descriptive cross-sectional study puts emphasis on comparison of the pattern of lipid profile in pre- and postmenopausal women. The study also highlights the atherogenic indices on the two groups.

Out of total 260 healthy women, 130 were premenopausal women aged from 22 to 50 years and 130 were postmenopausal women aged from 45 to 85 years. Serum TC, TAG, HDL-C, and LDL-C levels were found to be statistically significantly increased in postmenopausal women in comparison with premenopausal women (*P* value < 0.05) in our study. Almost all study shows increased levels of TC, TAG, and LDL-C in postmenopausal group compared to premenopausal group but there is a wide controversy regarding changes in HDL-C after menopause. Our result was quite similar to the study conducted by Ifueko (2013), in which TC, TAG, HDL-C, and LDL-C were increased significantly with “*P*” value < 0.001 in postmenopausal women than in premenopausal women [14]. Likewise, Shenoy and Vernekar (2015) also found significantly increased TC, TAG, and LDL-C but the HDL-C was not increased significantly in postmenopausal women in comparison to premenopausal women [15]. Similarly, a study done by Derby et al. (2009) showed a result of significant increase in TC and LDL-C while TAG was increased nonsignificantly and there was no changes in HDL-C in postmenopausal women when compared to premenopausal women [16]. A study done by Peters et al. in 1999 found significantly increased TC and LDL-C but the increase in TAG and HDL-C was nonsignificant in postmenopausal women than in premenopausal women [17]. Another similar study done by Berg et al. (2004) found nonsignificant increase in TC and TAG, no change in HDL-C, and significant increase in LDL-C in postmenopausal women when compared to premenopausal women [13]. Likewise, Igweh et al. in 2005 showed nonsignificant increase in TC and TAG, significant decrease in HDL-C, and significant increase in LDL-C in postmenopausal women when compared to premenopausal women [18]. Also a study done by Sapkota et al. in 2015 in Nepal showed significantly increased TC, TAG, and LDL-C which is in agreement with the present study and significantly decreased HDL-C in postmenopausal women than in premenopausal women which is in contrast to the present study [11].

A longitudinal study done by Derby et al. showed increasing trend of TC from premenopausal through menopausal transition to postmenopausal status [16]. Though various factors influencing cholesterol are prevalent, most detrimental cause of increase in TC level in postmenopausal women could be alteration in the cardioprotective sex hormone especially estrogen and progesterone. Also some studies have revealed the occurrence of great genetic variance for most lipids [3].

Though the level of TAG is increased in postmenopausal women few studies found that the increase was not significant. This variation in increasing pattern may be due to variation in study population and different age group of postmenopausal women recruited in the study. The possible reason behind the increase in TAG in postmenopausal women is the effect of estrogen on adipocyte cells and lipoprotein lipase activity. Estrogen deficiency will enhance the increase in adipocyte cell size and also increases the activity of lipoprotein lipase [3].

There is vast controversy in the level of HDL in postmenopausal women. Some studies revealed the significant decrease in level of HDL in postmenopausal women while some studies revealed significant increase in level of HDL in postmenopausal women supporting the present study. This discrepancy result may be due to variation in study population, life intervention, and duration of menopause. A longitudinal study done by Derby et al. showed there was gradual increase in HDL from premenopause through menopausal transition to postmenopause. However, there was slight decrease in HDL level at late postmenopause [16].

Most studies showed significant increase in LDL cholesterol level supporting the present study, without any contradiction. This obvious result observed in all studies may represent the fact that estrogen has direct effect on LDL metabolism. The main reason for the absolute increase in LDL may be due to the decrease in estrogen that stimulated the synthesis of LDL receptor which directly causes reduction of LDL receptor after menopause [3].

In our study, TC, TAG, and LDL-C were positively correlated with BMI while HDL-C was negatively correlated with BMI in postmenopausal women, which may indicate poor dietary and physical activity intervention among our postmenopausal population.

The atherogenic indices were obtained in the present study which showed TC/HDL with average of 3.5 in premenopause and 3.7 in postmenopause while another similar study done by Ifueko showed a contrast result with 2.68 and 2.39 in pre- and postmenopause, respectively. This shows that the present study population group is more at risk of having complications associated with cardiovascular disease. Furthermore, the LDL/HDL ratio in present study was found to be average of 1.9 and 2.2 in pre- and postmenopause, respectively, while the other similar study done by Ifueko found result of 0.95 and 0.84 in pre- and postmenopause, respectively, which was dissimilar to the present finding.

Though both HDL-C and LDL-C were found to be significantly increased in postmenopausal women as compared to premenopausal, significantly increased LDL/HDL ratio in postmenopausal women implied that atherogenic cholesterol, LDL-C, is aggregating more rapidly than good cholesterol, HDL-C. This indicates that the postmenopausal women in study population are more prone to cardiovascular risk.

## 5. Conclusion

Potentially, adverse changes in lipid profile along with significant increase in cardiac risk ratio in postmenopausal women of the study remark that this group of women is at increased risk of having complications associated with cardiovascular disease in near future. Early and timely detection and primary prevention can avoid morbidity and mortality in this high risk population. Moreover, positive correlation of BMI with TC and TAG in pre- and postmenopausal women concludes that dietary interventions and physical activity are to be encouraged in both the study groups especially when other cardiac risk factors are prevailing.

## Figures and Tables

**Table 1:** Lipid profile in pre- and postmenopausal women (*N* = 260).

	Premenopause	Postmenopause	*P* value
	Mean ± SD	Mean ± SD	
	(*N* = 130)	(*N* = 130)	
TC	148.9 ± 37.1	181.3 ± 43.6	<0.001
TAG	127.9 ± 71.4	148.2 ± 89.7	<0.05
HDL-C	43.0 ± 10.8	50.7 ± 14.4	<0.001
LDL-C	80.7 ± 32.1	104.4 ± 42.9	<0.001

TC: Total Cholesterol, TAG: Triacylglycerol, HDL-C: High Density Lipoprotein Cholesterol, and LDL-C: Low Density Lipoprotein Cholesterol.

**Table 2:** Cardiac risk ratio in study population (*N* = 260).

	Premenopause	Postmenopause	*P* value
	Mean ± SD	Mean ± SD	
	(*N* = 130)	(*N* = 130)	
TC/HDL	3.5 ± 1.1	3.7 ± 1.3	0.2
TAG/HDL	3.1 ± 2.0	3.2 ± 2.5	0.8
LDL/HDL	1.9 ± 0.8	2.2 ± 1.2	0.03

**Table 3:** Correlation of BMI with lipid profile parameters in pre- and postmenopausal women (*N* = 260).

	BMI			
	Premenopause		Postmenopause	
	(*N* = 130)		(*N* = 130)	
	*r*-value	*P* value	*r*-value	*P* value
TC	0.236^*∗∗*^	0.007	0.192^*∗*^	0.02
TAG	0.332^*∗∗*^	0.001	0.254^*∗∗*^	0.004
HDL	0.033	0.7	−0.155	0.07
LDL	0.132	0.13	0.167	0.057

^*∗*^Correlation being significant at 0.01 level

^*∗∗*^Correlation being significant at 0.05 level.

## Abbreviations

CVD: Cardiovascular disease
TAG: Triacylglycerol
VLDL: Very low density lipoprotein
LDL-C: Low Density Lipoprotein Cholesterol
HDL-C: High Density Lipoprotein Cholesterol
FSH: Follicle stimulating hormone
LH: Luteinizing hormone
BMI: Body mass index
LPL: Lipoprotein lipase
HSL: Hormone sensitive lipase.

## Additional Points

*Limitations*. This study was time framed study, conducted during the period of six months in a community of Lalitpur, Nepal, which limits the sample size to establishing causality of this descriptive cross-sectional study. This study could not give definite range of age group in both pre- and postmenopausal groups as samples were collected randomly from the specific community. In addition, the study also limits establishing the relation of cardiovascular risk with the social status of study population.
